# *OsPRR37* Alternatively Promotes Heading Date Through Suppressing the Expression of *Ghd7* in the *Japonica* Variety Zhonghua 11 under Natural Long-Day Conditions

**DOI:** 10.1186/s12284-021-00464-1

**Published:** 2021-02-25

**Authors:** Yong Hu, Xin Zhou, Bo Zhang, Shuangle Li, Xiaowei Fan, Hu Zhao, Jia Zhang, Haiyang Liu, Qin He, Qiuping Li, Mohammed Ayaad, Aiqing You, Yongzhong Xing

**Affiliations:** 1 Hubei Key Laboratory of Food Crop Germplasm and Genetic Improvement, Food Crops Research Institute, Hubei Academy of Agricultural Sciences, Wuhan, 430064 China; 2 National Key Laboratory of Crop Genetic Improvement and National Center of Plant Gene Research (Wuhan), Huazhong Agricultural University, Wuhan, 430070 China; 3 College of Agriculture, Yangtze University, Jingzhou, 434000 China; 4 Plant Research Department, Nuclear Research Center, Atomic Energy Authority, Abo-Zaabal, 13759 Egypt

**Keywords:** Rice, Heading date, Alternative function, MutMap, Regulatory pathway

## Abstract

Heading date is an important agronomic trait of rice (*Oryza sativa* L.) and is regulated by numerous genes, some of which exhibit functional divergence in a genetic background-dependent manner. Here, we identified a *late heading date 7* (*lhd7*) mutant that flowered later than wild-type Zhonghua 11 (ZH11) under natural long-day (NLD) conditions. Map-based cloning facilitated by the MutMap strategy revealed that *LHD7* was on the same locus as *OsPRR37* but exhibited a novel function as a promoter of heading date. A single-nucleotide mutation of G-to-A in the coding region caused a substitution of aspartic acid for glycine at site 159 within the pseudo-receiver (PR) domain of OsPRR37. Transcriptional analysis revealed that *OsPRR37* suppressed *Ghd7* expression in both ZH11 background under NLD conditions and the Zhenshan 97 background under natural short-day conditions. Consistently, the expression of *Ehd1*, *Hd3a* and *RFT1* was enhanced by *OsPRR37* in the ZH11 background. Genetic analysis indicated that the promotion of heading date and reduction in grain yield by *OsPRR37* were partially dependent on *Ghd7*. Further investigation showed that the alternative function of *OsPRR37* required an intact Ghd7-related regulatory pathway involving not only its upstream regulators *OsGI* and *PhyB* but also its interacting partner Hd1. Our study revealed the distinct role of *OsPRR37* in the ZH11 background, which provides a more comprehensive understanding of *OsPRR37* function and enriches the theoretical bases for improvement of rice heading date in the future.

**Supplementary Information:**

The online version contains supplementary material available at 10.1186/s12284-021-00464-1.

## Background

The heading date (also known as flowering time) of rice is defined as the time from sowing date to the emergence of the first panicle. It largely determines the regional and seasonal adaptation of a specific variety. A complicated regulatory network of heading date in rice has been elucidated during the last two decades, among which *Oryza sativa Pseudo-Response Regulator37* (*OsPRR37*), also known as *Grain Number, Plant Height, and Heading Date7.1* (*Ghd7.1*)/*Days to heading 7* (*DTH7*)/*Heading date 2* (*Hd2*) (hereafter referred to as *OsPRR37*) is one of the most important components (Gao et al. 2014; Koo et al. 2013; Lin et al. 2000; Yan et al. 2013).

*OsPRR37* encodes a pseudo-response regulator (PRR) protein that contains an N-terminal pseudo-receiver (PR) domain and a C-terminal CCT (CONSTANS, CO-like, and TOC1) domain. A previous study demonstrated that *OsPRR37* delays heading date, increases plant height and enhances grain yield (Gao et al. 2014; Liu et al. 2015; Yan et al. 2013). Genetic diversity analysis revealed various non-functional variants including two frameshift mutations at 1515 bp and 1551 bp of the coding region between the PR and CCT domains, a gain of stop codon mutation at 2113 bp of the coding region in the CCT domain and a missense mutation at L710P in the CCT domain (Gao et al. 2014; Koo et al. 2013; Yan et al. 2013). In addition, other variants in the PR domain and the region between the PR and CCT domains were also detected, but their effects on the function of OsPRR37 were not demonstrated (Gao et al. 2014; Koo et al. 2013; Yan et al. 2013).

Natural variation analysis revealed that there are a total of 24 haplotypes of *OsPRR37* among 178 cultivated rice varieties and 47 wild rice (*O. rufipogon*) accessions. Five functional haplotypes of *OsPRR37* in cultivated varieties were also found in wild rice, but six rare defective haplotypes were not found, which suggested the pre-existence of genetic variations in wild rice accessions and the acquisition of mutations during domestication progression (Yan et al. 2013). Functional alleles of *OsPRR37* were mainly found in central and southern China, while weak and defective alleles were found in areas from central to northern China (Yan et al. 2013). This distinct eco-geographical distribution pattern of *OsPRR37* was further supported by the finding that *japonica* varieties harboring nonfunctional alleles of both *OsPRR37* and *Ghd7* flower extremely early and are adapted to the northernmost regions (Koo et al. 2013; Li et al. 2015; Ye et al. 2018). In addition to the contribution of *OsPRR37* alone to the regional and seasonal adaptation of cultivars, gene combinations of *OsPRR37*, *Ghd7* and *Ghd8* were highly correlated with grain yield under different photoperiod conditions (Gao et al. 2014; Zhang et al. 2019a).

The transcriptional regulation of downstream targets of *OsPRR37* has been reported. Some studies have shown that *OsPRR37* suppresses the expression of *Early heading date 1* (*Ehd1*) and thus suppresses *Heading date 3a* (*Hd3a*) and *RICE FLOWERING LOCUS 1* (*RFT1*) (Gao et al. 2014; Yan et al. 2013), while other studies have suggested that *OsPRR37* directly downregulates the expression of *Hd3a* (Koo et al. 2013). *OsPRR37* acts downstream of rice *Phytochrome B* (*PhyB*) because mutation of *phyB* largely dampens the expression level of *OsPRR37* under both long-day (LD) and short-day (SD) conditions (Gao et al. 2014). *OsPRR37* is an ortholog of *Arabidopsis PRR7* that is a crucial component of the circadian circuit. The functional allele of *OsPRR37* complements the late-flowering phenotype of the *prr7* mutant, which supports the conservative function between these two genes (Koo et al. 2013).

In addition to *OsPRR37*, other regulators such as *Ghd7* and *Hd1* also play important roles in the regulation of heading date. Ghd7 is another CCT domain-containing protein that acts as a strong heading date suppressor under LD conditions. The expression of *Ghd7* is positively regulated by *Oryza sativa GIGANTEA* (*OsGI*) (Itoh et al. 2010), an ortholog of *Arabidopsis GI*, which acts as an output regulator of the circadian clock (Mizoguchi et al. 2005). *OsGI* is essential for setting the critical day length for the expression level of *Hd3a* by regulating *Ghd7* and *Ehd1* (Itoh et al. 2010). In addition, *OsELF3* and *Ehd3* were also reported as upstream regulators of *Ghd7* (Matsubara et al. 2012; Matsubara et al. 2011; Saito et al. 2012; Yang et al. 2013). *PhyB*, a rice red/far-red light receptor, is involved in either transcriptional (Osugi et al. 2011) or post-transcriptional regulation of *Ghd7* (Weng et al. 2014). Genetic analysis revealed the additive effects of *Ghd7* and *OsPRR37*, which suggested their independent roles in the heading date regulation (Koo et al. 2013). *Hd1*, an ortholog of *Arabidopsis CONSTANS*, exhibits divergent and more complicated functions in heading date regulation in rice (Yano et al. 2000). *Hd1* promotes heading date under SD conditions, but exhibits distinct effects under LD conditions. In the background with non-functional alleles of *Ghd7* and *OsPRR37*, *Hd1* consistently promotes heading date. However, alternative genetic effects are switched by combination with the functional allele of *Ghd7* or *OsPRR37* (Fujino et al. 2019; Subudhi et al. 2018; Zhang et al. 2019a; Zhang et al. 2017; Zhang et al. 2019b). Further study demonstrated that Hd1 interacts with Ghd7 and forms a complex that specifically binds to the *cis*-regulatory region in the *Ehd1* (Nemoto et al. 2016).

In this study, *OsPRR37* was found to exhibit alternative functions as a promoter of heading date in the ZH11 background under natural long-day (NLD) conditions. Transcriptional analysis revealed that *OsPRR37* suppressed *Ghd7* expression in both the ZH11 background under NLD conditions and the Zhenshan 97 background under natural short-day (NSD) conditions. Genetic analysis further revealed that the promotion of heading date by *OsPRR37* partially relies on an intact *Ghd7*-related pathway involving not only its upstream regulators *OsGI* and *PhyB*, but also the Ghd7 interacting protein Hd1.

## Materials and Methods

### Plant Materials

The *lhd7* (*osprr37*) mutant was identified in the M_2_ generation of an ethyl methane sulfonate-treated *japonica* rice cultivar, Zhonghua 11 (ZH11, *Oryza sativa* L.). The mutant of *ghd7* with a G to A mutation in the coding region of variety ZH11 resulting in a premature stop codon was described in our previous study (Hu et al. 2019). Using the CRISPR method, *OsGI*, *PhyB* and *Hd1* were individually knocked out in the both the ZH11 and *osprr37* mutant backgrounds, and *Ghd7* was knocked out in the *osprr37* mutant background. The near-isogenic lines (NILs) *Ghd7 Ghd8 Hd1*, *Ghd7 Ghd8 hd1*, and *Ghd7 ghd8 Hd1* were generated in the previous study (Zhang et al. 2019a). Briefly, these NILs were segregated and selected in a NIL-F_2_ population deriving from the NIL-F_1_ generated by crossing two NILs in the Zhenshan 97 (ZS97, *Oryza sativa* L. ssp. *indica*) background, NIL1 (*Ghd7 Ghd8 osprr37 Hd1*) and NIL2 (*ghd7 ghd8 OsPRR37 hd1*).

### Plant Growth Conditions

The rice plants were examined under NLD (day length more than 13.5 h) conditions from mid-May to August in Wuhan (Huazhong Agricultural University, 114°21′ E, 30°28′ N) or NSD (day length less than 12.5 h) conditions from December to April in Lingshui, Hainan (110°2′ E, 18°30′ N). The plants used for expression analysis of flowering time genes were grown in chambers with controlled environment under LD (14 h light/10 h dark) conditions. Heading dates under NLD and NSD conditions were scored as the number of days from germination to the emergence of the first panicle.

### MutMap Analysis of Heading Date Gene

The MutMap strategy (Abe et al. 2012) with some modifications was applied for map-based cloning of *LHD7*. Fifty extremely late heading individuals of the F_2_ population were bulked and sequenced with an Illumina HiSeq 1000 instrument. Sequence reads were filtered using Trimmomatic (version 0.36) (Bolger et al. 2014). Then the clean data were mapped against the MSU 7.0 rice genome with corresponding annotation by BWA (version 0.7.17) (Li and Durbin 2009), and sorted with SAMtools (version 1.8) (Li et al. 2009). These data were then analyzed with GATK (version 3.8) for variant calling (McKenna et al. 2010). The SNP index defined as the ratio of the number of reads of a mutant SNP to the total number of reads corresponding to the SNP (Abe et al. 2012) was calculated for each SNP. Finally, R-CMplot (https://github.com/YinLiLin/R-CMplot/) was used for visualization of the absolute value of Δ (SNP index) which indicates the difference in the SNP index between the bulked pool and ZH11.

### Kompetitive Allele Specific PCR (KASP) Assays

In the progeny test, the KASP assay was used for genotyping SNPs in each individual plant. SNP-specific primers (Additional file 1: Table S1) were designed online at http://www.snpway.com/. For each reaction, 5–50 ng DNA of a specific individual was used in a total reaction volume of 5 μL, that contained 2.5 μL of KASP Master Mix (LGC Biosearch Technologies, Petaluma, California, USA), 0.075 μL (100 μM) of two allele specific primers and 2 μL (100 μM) of common primer. The PCR conditions were as follows: denaturation at 94 °C for 15 min followed by 10 cycles of 20 s at 94 °C and 1 min at 65–57 °C (decreasing 0.8 °C per cycle), followed by another 41 cycles of 20 s at 94 °C and 1 min at 57 °C. Once the thermal cycling was complete, the plates containing the PCR reactions were read with a BMG FLUOstar Omega (LGC Biosearch Technologies, Petaluma, California, USA). Finally, the data were analyzed with KlusterCaller software (LGC Biosearch Technologies, Petaluma, California, USA).

### Vector Construction and Transformation

The coding sequence of *OsPRR37* was amplified by PCR using the primers Ghd7.1-UF and Ghd7.1-UR (Additional File 1: Table S1) and inserted in into pU1301 with a Gibson assembly reaction (Gibson et al. 2009). To construct the CRISPR-Cas9 vector for *Ghd7*, the target sequence was designed online (http://crispr.hzau.edu.cn/CRISPR2/ (Lei et al. 2014)) and fused to the Ghd7-CRF and Ghd7-CRR primers (Additional File 1: Table S1). With a segment-overlapping PCR followed by a Gibson assembly reaction, the target sequence with a U3 promoter sequence was cloned into a pCXUN-Cas9 vector (He et al. 2017; He et al. 2018). Other CRISPR-Cas9 constructs for *OsGI*, *PhyB* and *Hd1* were generated by the same method with corresponding primers (Additional File 1: Table S1). These vectors were induced into indicated acceptors with *Agrobacterium*-mediated transformation (Hiei et al. 1994).

### RNA Sampling and Gene Expression Analysis

To analyze the transcriptional effects of *OsPRR37* on other heading date genes, ZH11 and *osprr37* mutant were grown under controlled LD conditions (14 h light/10 h dark). The leaves from 35-d-old plants were sampled every 4 h within a 24-h period, and three different individuals per time point were used as biological replicates. Total RNA was isolated with TRIzol reagent (Transgen Biotech, Beijing, China). For reverse transcription quantitative PCR (RT-qPCR), first-strand cDNA was synthesized using reverse transcriptase (Invitrogen), and qPCR was then performed using gene-specific primers (Additional File 1: Table S1), SYBR Master Mix reagent (Roche), and a Quant-Studio 6 Flex Real-Time PCR System (Life Science), according to the manufacturer’s instructions. The PCR conditions were as follows: 10 min at 95 °C followed by 40 cycles of 10 s at 95 °C and 30 s at 60 °C. PCR amplifications were conducted in triplicate for each sample from three independent biological replicates, and a rice ubiquitin gene (Os02g0161900) was used for normalization.

### Protein Sequence Alignments

Alignments were conducted with ClustalX (version 2.1) by using protein sequences of OsPRR37 (from ZH11 type), OsPRR73 (BAD38856 in DDBJ/EMBL/GenBank), OsPRR95 (BAD38857), OsPRR59 (AK120059), OsTOC1 (BAD38854) from *Oryza sativa*; PtPRR37 (XP_002311123.1), PtPRR73 (XP_002316333.1), PtPRR9a (XP_002320232.1), and PtPRR9b (XP_002301443.1) from *Populus trichocarpa*; AtPRR3 (BAB13744), AtPRR5 (BAB13743), AtPRR7 (BAB13742), AtPRR9 (BAB13741), and AtTOC1 (NP_200946) from *Arabidopsis thaliana*; and PpPRR1 (AB558266), PpPRR2 (AB558268), PpPRR3 (AB558267), and PpPRR4 (AB558269) from *Physcomitrella patens*.

## Results

### Phenotype of *Late Heading Date 7* (*lhd7*) Mutant

**Fig. 1:**
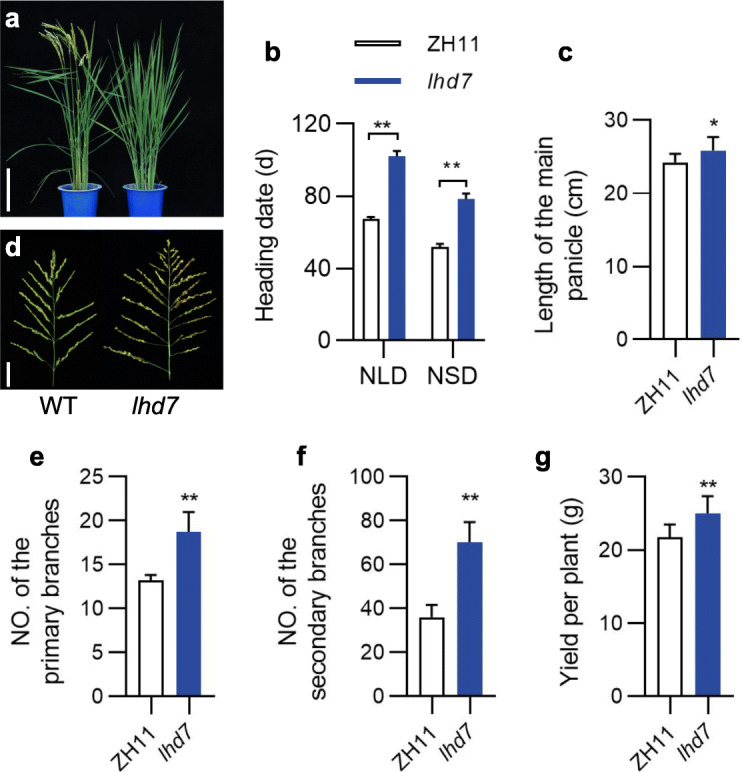
Phenotype of *lhd7* mutant compared with Zhonghua 11 (ZH11, *Oryza sativa* L. ssp. *japonica*). **a** Phenotype of whole plants of *lhd7* and ZH11 grown under natural long-day (NLD) conditions. Scale bar, 25 cm. **b** Heading date performances of *lhd7* and ZH11 under both NLD and natural short-day (NSD) conditions**. c-g** Comparison of length of the main panicles (**c**), phenotype of main panicle (**d**), number of primary branches (**e**), number of secondary branches (**f**), and yield per plant (**g**) between *lhd7* mutant and ZH11 grown under NLD conditions. Data represent the mean ± standard deviation (SD), *, *P* < 0.05; **, *P* < 0.01; *t*-test, *n* = 16; Scale bar in B, 5 cm

The late heading date mutant *lhd7* was identified from the M_2_ plants of ethyl methane sulfonate (EMS)-treated rice cultivar ZH11. The *lhd7* mutants (102.1 ± 3.0 d) flowered about 35 d later than that of control ZH11 plants (67.4 ± 1.0 d) under both natural long-day (NLD) and natural short-day (NSD) conditions (Fig. 1a, b). The *lhd7* mutant exhibited longer and denser panicles (Fig. 1c, d) with more primary (Fig. 1e) and secondary branches (Fig. 1f). No significant difference (*p* = 0.16, *t*-test) of effective panicles was observed between *lhd7* (8.1 ± 0.6) and WT (7.6 ± 0.5). Additionally, the *lhd7* plants were found producing more yield per plant than that of ZH11 plants under NLD conditions (Fig. 1g).

### Cloning of *LHD7* with MutMap Strategy

**Fig. 2:**
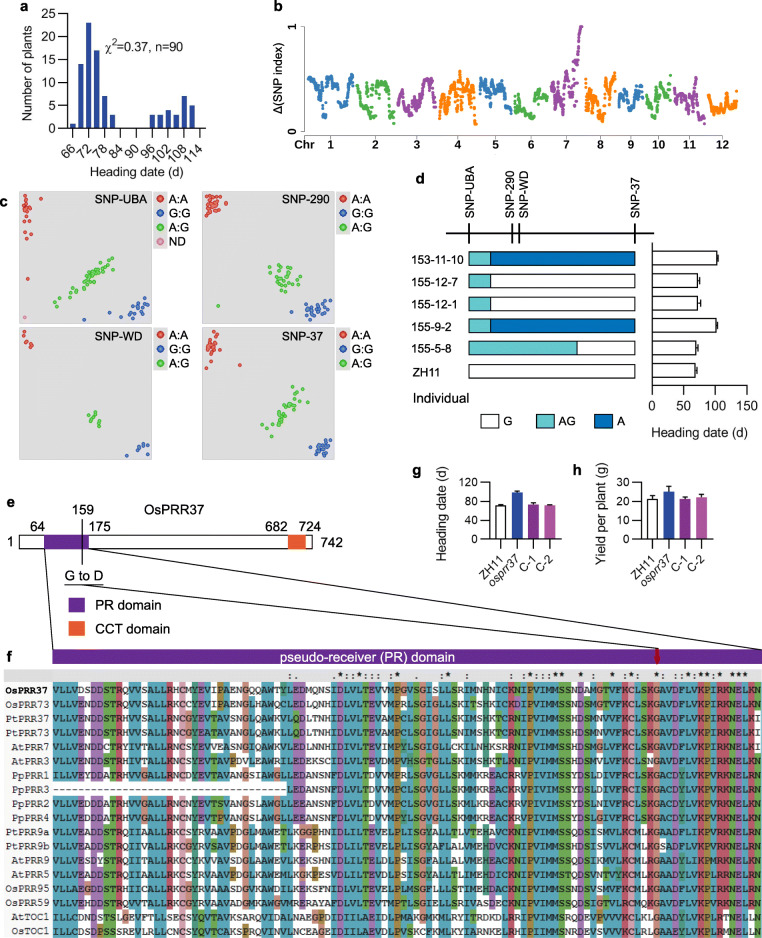
Map-based cloning of *LHD7*. **a** Chi-square test of heading-date distribution of an F_2_ population derived from the cross between *lhd7* mutant and ZH11. **b** Distribution of Δ index of single nucleotide polymorphism (SNP) across 12 chromosomes. Δ (SNP index) means the absolute value of the difference of SNP index between the bulked pool and ZH11. **c** Genotyping examples with SNP markers used in kompetitive allele specific PCR (KASP) assay of the F_2_ population. Dots in different colors and grouped together represent different genotype of the SNPs as indicated. **d** Progeny tests of 5 recombinants. Numbers of individual plant and information of SNP markers were listed on the left and above sides, respectively. Column chart on the right showed the phenotype of heading date of each plant. Bars with different colors represent different genotype as legends indicated below. **e** Protein structure and detailed mutation of *OsPRR37* in *lhd7* mutant. Bars with different colors represent different protein domains of OsPRR37 as indicated. Numbers near the bars represent the position of amino acid. **f** Conservation of pseudo-receiver (PR) domain of OsPRR37. Aliments of PR domain of Pseudo responsive regulator (PRR) proteins from different organisms. Red arrow indicates the amino acid Gly (G) at position 159 which mutated to Asp (D) in osprr37. **g-h** Phenotype of heading date and yield per plant of complementary lines compared with ZH11 and *lhd7* mutant

The *lhd7* mutant was backcrossed with ZH11 to produce an F_2_ population in which the number of early heading plants to that of later heading plants fit a ratio of 3:1 (chi square = 0.37, df = 1, *p* = 0.54) (Fig. 2a). This result indicated that the variation in heading date is controlled by a single gene (Fig. 2a). Fifty extremely late heading individuals of the F_2_ population were bulked and used for MutMap analysis with next-generation resequencing (Abe et al. 2012). The single nucleotide polymorphism (SNP) indexes were calculated for each SNP. A distinct Δ (SNP index) peak of 1 harboring a cluster of four SNPs was detected at the end of the long arm of chromosome 7 (Fig. 2b, Table 1). To further analyze the causal SNP associated with the *lhd7* phenotype, a kompetitive allele specific PCR (KASP) assay was applied to the F_2_ population with 498 individuals (Fig. 2c), within which five recombinants between marker SNP-UBA and SNP-37 were identified (Fig. 2d, Additional File 1: Table S1). Progeny tests of these recombinants showed a co-segregation relationship between heading-date phenotypes and marker genotypes of SNP-37 (Fig. 2c), which is located in the coding sequence of *OsPRR37* that was previously identified as a strong suppressor of heading date (Gao et al. 2014; Koo et al. 2013; Yan et al. 2013). The G to A mutation at SNP-37 caused an amino acid substitution of glycine (G) to aspartic acid (D) at position 159 (G159D) within the PR domain of the OsPRR37 protein (Fig. 2e). Interestingly, the amino acid glycine at site 159 was highly conserved among PRR7 homologs in different organisms (Fig. 2f). To further validate that *LHD7* was allelic to *OsPRR37*, a fragment containing the full-length coding region of *OsPRR37* from ZH11 under the control of a maize *ubiquitin* promoter was transformed into the *lhd7* mutant. Both the heading date and yield per plant of transgenic plants were restored to the level of ZH11 under NLD conditions (Fig. 2g, h). Thus, *LHD7* was *OsPRR37* (hereafter referred to as *OsPRR37*) but exhibited an alternative function as a promotor of heading date in the ZH11 background under NLD conditions.

**Table 1:** Information of SNPs with Δ (SNP index) of 1

POS	REF	ALT	Δ (SNP index)	LOC
29,480,375	G	A	1.00	LOC_Os07g49230
29,517,850	G	A	1.00	LOC_Os07g49280
29,523,655	G	A	1.00	LOC_Os07g49300
29,623,477	G	A	1.00	LOC_Os07g49460

POS, the physical position of indicated SNP; REF, the allele of ZH11 of indicated SNP; ALT, alternative allele in the bulked pool of indicated SNP; Δ (SNP index), the absolute value of difference between ZH11 and bulked pool of the SNP index as the ratio between the number of reads of a mutant SNP and the total number of reads corresponding to the SNP; LOC, locus of SNP located in

### *OsPRR37* Suppresses the Expression of *Ghd7* in the ZH11 and Zhenshan 97 Backgrounds under Different Day-Length Conditions

**Fig. 3:**
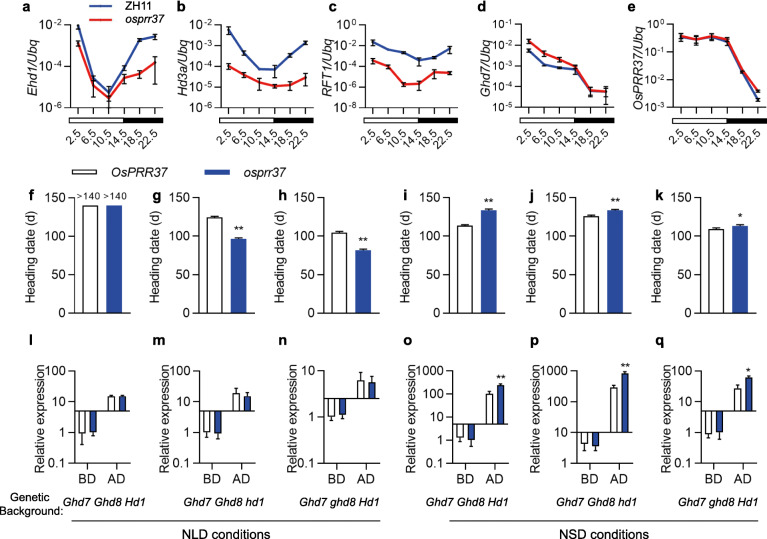
Expression analysis of heading date genes in *osprr37* mutant and different NILs. **a-e** Log_10_-transforemed expression levels of indicated genes in leaves of 40-d-old plants under controlled LD conditions were determined by quantitative real-time PCR (qRT-PCR) and shown as mean ± SD of three replicates. Rice ubiquitin gene (Os02g0161900) was used for normalization. **f-k** Comparing of heading date of near isogenic lines of *OsPRR37* in Zhenshan 97 background harboring different alleles of *Ghd7*, *Ghd8* and *Hd1* under NLD (**f-h**) and NSD (**i-k**) conditions. **i-q** Comparing of *Ghd7* expression in near isogenic lines indicated in **f-k** under controlled environments. Expression level of *Ghd7* were detected 2.5 h before (BD) and after dawn (AD) and shown as Log_10_-transforemed style. Leaves of 40-d-old plants under indicated conditions was collected and used for quantitative real-time PCR (qRT-PCR). Data was shown as mean ± SD of three replicates. Rice ubiquitin gene (Os02g0161900) was used for normalization. *, *P* < 0.05; **, *P* < 0.01; *t*-test

To further analyze the pathway through which *OsPRR37* was involved in promoting heading date, the expression levels of multiple heading-date related genes were compared between the *osprr37* mutant and ZH11. As expected, the expression of the flowering time integrator *Ehd1* (Fig. 3a), and two florigen genes, *Hd3a* (Fig. 3b) and *RFT1* (Fig. 3c), was strongly suppressed in the *osprr37* mutant, especially at zeitgebers 2.5 and 22.5 when they were highly expressed in ZH11. Interestingly, the expression of *Ghd7* at zeitgeber 2.5 was enhanced in the *osprr37* mutant compared with ZH11 (Fig. 3d). Comparable expression patterns and levels of other flowering genes including *OsPRR37* itself (Fig. 3e), *Hd1*, *Ghd8*, *OsGI*, *OsELF3*, *OsMADS50*, *OsMADS51*, *OsMADS56*, *Oryza sativa CONSTANS 3* (*OsCO3*), *CONSTANS LIKE 4* (*COL4*), *OsCOL10*, *OsCOL13*, *DTH2*, *Ehd2*, *Ehd3*, and *Ehd4* were observed between *osprr37* and ZH11 (Additional file 2: Figure S1). Moreover, in the background of Zhenshan 97, *OsPRR37* showed distinct functions in different day-length conditions. *OsPRR37* promoted heading date under NSD conditions, as in ZH11, but delayed heading date under NLD conditions (Fig. 3f-k). The expression of *Ghd7* was also found to be significantly enhanced after dawn in near-isogenic lines harboring defective *osprr37* under NSD but not NLD conditions (Fig. 3i-q). Together, these results implied that *OsPRR37* may promote flowering through suppressing the expression level of *Ghd7*.

### Heading Date Promotion by *OsPRR37* Required Functional *Ghd7*

**Fig. 4:**
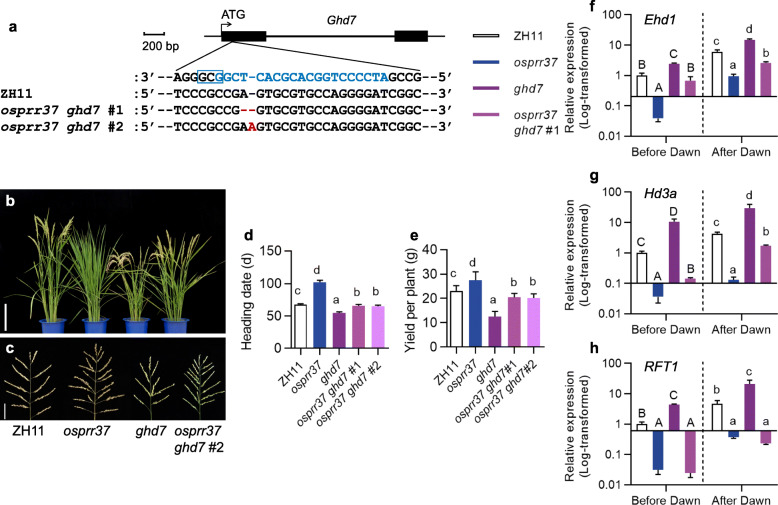
Genetic analysis between *OsPRR37* and *Ghd7*. **a** Allele information of *ghd7* mutant generated by clustered regularly interspaced short palindromic repeats (CRISPR) strategy. Black bars represent the exons, and solid line before, between and after the black bars represents the 5′ un-transcribed region, intron and 3′ un-transcribed region, respectively. Letters in the blue box is the protospacer adjacent motif (PAM) sequence. Blue letters indicate the targets sequence. Red letters indicate the mutation details. **b-c** Phenotype of whole plants (**b**) and main panicles (**c**) of ZH11, *osprr37*, *ghd7* and double mutants *osprr37 ghd7* (#1 and #2) grown under NLD conditions. Scale bar, 25 cm in **b** and 5 cm in **c**. **d-e** Heading date and yield per plant of ZH11, *osprr37*, *ghd7*, and the double mutants *osprr37 ghd7* (#1 and #2) grown under NLD conditions. Data represent mean ± SD, *n* = 10. Different letters indicate significant differences, Duncan’s test. **f-h** Log_10_-transformed expression levels of *Ehd1* (**f**), *Hd3a* (**g**) and *RFT1* (**h**) in ZH11, *osprr37*, *ghd7* and the double mutant *osprr37 ghd7*. Expression of indicated genes in leaves of 40-d-old plants under controlled LD conditions were determined 2.5 h before and after dawn by qRT-PCR and shown as mean ± SD of three replicates. Rice ubiquitin gene (Os02g0161900) was used for normalization. Different upper- and lower-case letters indicate significant differences at *P* < 0.05 by using Duncan’s test to compare the expression levels of indicated genes before and after dawn, respectively

To analyze the relationship between *OsPRR37* and *Ghd7*, we obtained the *osprr37 ghd7* double mutant through knocking out *Ghd7* in the *osprr37* background with clustered regularly interspaced short palindromic repeats (CRISPR) strategy (Fig. 4a-c). The *osprr37 ghd7* double mutants (#1: 65.8 ± 2.0 d, #2: 65.0 ± 1.6 d) flowered significantly earlier than the *osprr37* mutant (102.1 ± 3.0 d), but later than the *ghd7* mutant (54.7 ± 1.6 d) (Fig. 4d). The grain yield per plant of the *osprr37 ghd7* double mutants (#1: 20.5 ± 1.6 g, #2: 20.0 ± 1.64 g) appeared to be intermediate between single mutants *osprr37* (25.4 ± 4.2 g) and *ghd7* (12.6 ± 2.1 g) (Fig. 4e). To gain further insight into the relationship between *OsPRR37* and *Ghd7*, the expression levels of key genes in the regulation of heading date were investigated. At both testing points before and after dawn, the expression of *Ehd1* showed distinct levels in these lines with the lowest in *osprr37*, the highest in *ghd7* and the intermediate level in the double mutant *osprr37 ghd7* (Fig. 4f). *Hd3a* and *RFT1* showed similar expression patterns to *Ehd1* in these lines (Fig. 4g, h), except that comparable expression of *RFT1* was detected in *osprr37* and *osprr37 ghd7*. The differences in the expression levels of these key flowering genes are consistent with that of the phenotypic changes. Thus, the promotion of heading date by *OsPRR37* is partially attributed to the suppression of *Ghd7* expression in the ZH11 background.

### Effects of *OsGI*, *PhyB* and *Hd1* on the Function of *OsPRR37*

**Fig. 5:**
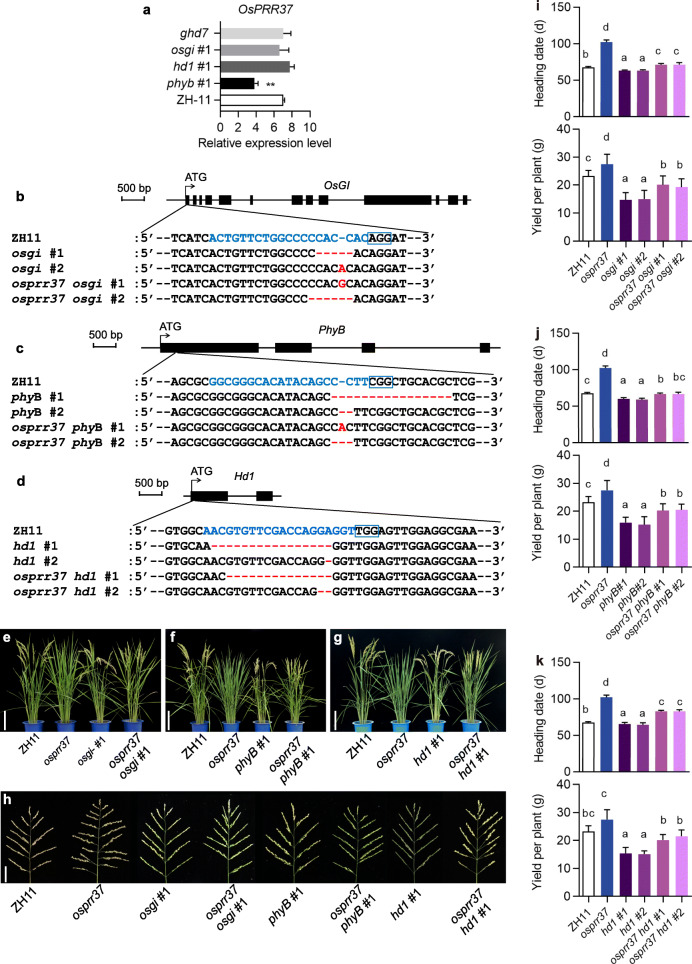
Generation of knockout mutants for *OsGI*, *PhyB* and *Hd1* with CRISPR strategy. **a** Comparison of expression of *OsPRR37* among mutants *ghd7*, *osgi*, *hd1*, *phyB*, and control plant ZH11. Expression level of *OsPRR37* in leaves collected 2.5 h after dawn from 40-d-old plants under controlled LD conditions were analyzed by qRT-PCR and shown as mean ± SD of three replicates. Rice ubiquitin gene (Os02g0161900) was used for normalization. **, *P* < 0.01, *t*-test. **b-d** Mutation details of genetic materials of *OsGI* (**b**), *PhyB* (**c**) and *Hd1* (**d**). Black bars represent the exons, and solid line before, between and after the black bars represents the 5′ un-transcribed region, intron and 3′ un-transcribed region, respectively. Letters in the blue box is the PAM sequence. Blue letters indicate the targets sequence of indicated gene. Red letters indicate the mutation details. **e-h** Phenotype of whole plants (**e-g**); and main panicles (**h**) of indicated genetic materials. Scale bar, 25 cm in **e**-**g** and 5 cm in **h**. **i-k** Genetic analyses between *OsPRR37* and *OsGI* (**i**), *PhyB* (**j**) and *Hd1* (**k**). Data represent mean ± SD, *n* = 16. Different letters indicate significant differences at *P* < 0.05, Duncan’s test

**Fig. 6:**
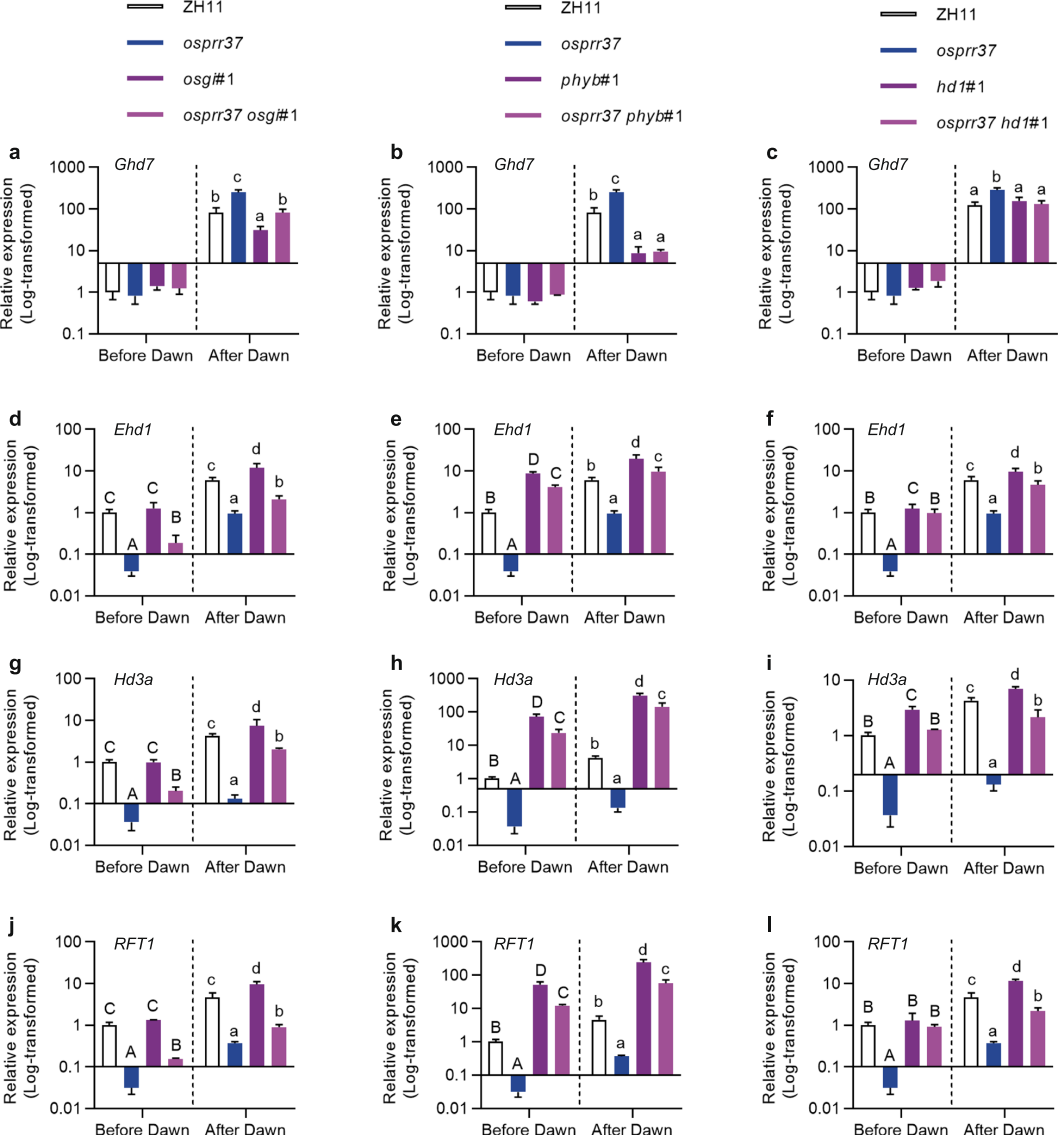
Expression analysis of key flowering genes in genetic materials related to *OsGI*, Os*PhyB* and *Hd1*. Log_10_-transformed expression of *Ghd7*, *Ehd1*, *Hd3a*, *and RFT1* in the single mutants and double mutants of *OsPRR37* and *OsGI* (**a, d, g** and **j**), *OsPRR37* and *PhyB* (**b, e, h** and **k**), and *OsPRR37* and *Hd1* (**c, f, i** and **l**), respectively. Samples were collected 2.5 h before and after dawn from 40-d-old plants under controlled LD conditions. Data represent mean ± SD of three replicates. Rice ubiquitin gene (Os02g0161900) was used for normalization. Different upper- and lower-case letters indicate significant differences at *P* < 0.05 by using Duncan’s test to compare the expression levels of indicated genes before and after dawn, respectively

The function of Ghd7 depends on intact elements involved in the *Ghd7*-related pathway including its upstream transcriptional regulators *OsGI* and *PhyB* and its physical interaction partner Hd1 (Itoh et al. 2010; Koo et al. 2013; Nemoto et al. 2016; Zhang et al. 2017). To validate the involvement of *Ghd7* in the regulation of heading date by *OsPRR37*, single mutants of *Ghd7*-related genes (*OsGI*, *PhyB* and *Hd1*) and double mutants of *OsPRR37* and these related genes were generated with the CRISPR method. *OsPRR37* expression in the *phyB* mutant was significantly reduced as compared with that in ZH11, but no decline was detected in the *osgi*, *hd1* or *ghd7* mutants (Fig. 5a). Moreover, *osgi* (Fig. 5b), *phyB* (Fig. 5c) and *hd1* (Fig. 5d) all headed earlier and exhibited decreased yield per plant compared with ZH11 under NLD conditions (Fig. 5e-k). Additionally, double mutants generated by knocking out *OsGI* (*osprr37 osgi*, Fig. 5b), *PhyB* (*osprr37 phyB*, Fig. 5c) and *Hd1* (*osprr37 hd1*, Fig. 5d) in the *osprr37* background displayed significantly earlier heading dates than the *osprr37* mutant alone but delayed heading dates compared with their corresponding single mutants, *osgi*, *phyB* and *hd1*, respectively (Fig. 5i-k). Significant declined expression of *Ghd7* at 2.5 h after dawn were detected in the *osgi* and *phyB* mutants but not the *hd1* mutant (Fig. 6a-c). Also, at 2.5 h after dawn, an intermediate expression level of *Ghd7* was observed in the *osprr37 osgi* double mutant compared with the *osprr37* and *osgi* single mutants (Fig. 6a), while the *osprr37 phyB* double mutant displayed a comparable *Ghd7* level to the *phyB* mutant (Fig. 6b). Mutation of *Hd1* did not affect the expression of *Ghd7* (Fig. 6c). Consistent with the phenotypic changes, the expression levels of the key heading date genes *Ehd1*, *Hd3a* and *RFT1* in the *osprr37 osgi*, *osprr37 phyB* and *osprr37 hd1* double mutants at 2.5 h after dawn were all higher than those in the *osprr37* single mutant but lower than those in corresponding single mutants *osgi*, *phyB* and *hd1*, respectively (Fig. 6d-l). These results together suggested that the promotion of heading date by *OsPRR37* requires an intact regulatory pathway of Ghd7 involving not only the upstream regulators *OsGI* and *PhyB*, but also the Ghd7-interacting protein Hd1.

## Discussion

### Alternative Function of *OsPRR37* Is Partially Relying on *Ghd7*

In this study, the delay of heading date in the *osprr37* mutant was due to the G159D mutation in the PR domain, which was highly conserved among its homologs in different organisms (Fig. 2e, f). PR domain is crucial for the function of PRR proteins. Through PR domain, PRR1/TOC1 interacts with ZTL, which targets PRR1/TOC1 for proteasome-dependent degradation (Kiba et al. 2007). In addition, PRR proteins could interact with each other and form heterodimers through their PR domains (Ito et al. 2003); this dimerization stabilizes the PRR protein and protects it from ZTL-dependent degradation (Para et al. 2007). Thus, it is possible that the G159D mutation might affect the interactions between OsPRR37 and other PRRs and the turnover of PRR proteins at the post-transcriptional level.

Our results demonstrated that *OsPRR37* promotes heading date and decreases grain yield in the ZH11 background (Fig. 4d, e), in contrast to previous reports that *OsPRR37* delays heading date and increases grain yield in genetic backgrounds of Zhenshan 97, Kita-ake, Milyang 23 and Dongjin under LD or NLD conditions (Gao et al. 2014; Koo et al. 2013; Yan et al. 2013). Similar effects of *OsPRR37* on promoting heading date were also found in the Zhenshan 97 background but only under NSD conditions (Fig. 3i-k). This dual role of OsPRR37 is similar to that of Hd1, which promotes heading date under LD conditions in the *ghd7*-defective; otherwise, it delays heading date by interacting with Ghd7 and directly suppressing the expression of *Ehd1* (Nemoto et al. 2016; Subudhi et al. 2018; Zhang et al. 2017). Apart from *Ghd7*, *OsPRR37* also switches the effect of *Hd1*, which was revealed by analyzing the genetic effects of combinations of *Hd1* and *OsPRR37* in a segregating population (Fujino et al. 2019). Considering the dual role of Hd1 in heading date regulation, Hd1 might be the factor that converts the function of OsPRR37. To test this, genetic analysis was performed between *OsPRR37* and *Hd1*. However, the *osprr37 hd1* double mutant headed later than the *hd1* single mutant (Fig. 5k), which went against with our expectation. Thus, *Hd1* is not the converting factor.

*Ghd7* expression was increased in the *osprr37* mutant in the ZH11 background under NLD conditions (Fig. 3d) and near-isogenic lines with defective *osprr37* in the Zhenshan 97 background under NSD conditions (Fig. 3o-q). Regardless of the day-length difference, these results together implied that *OsPRR37* acts upstream of *Ghd7*. The *osprr37 ghd7* double mutant in the ZH11 background showed significantly earlier heading date and reduced grain yield compared with the *osprr37* single mutant (Fig. 4b-e), which further confirmed the involvement of *Ghd7* in the pathway of heading date regulation by *OsPRR37*. However, the *osprr37 ghd7* double mutant still headed later and produced more grains than the *ghd7* single mutant (Fig. 4d-e), which suggested that the promotion of heading date by *OsPRR37* is partially dependent on *Ghd7* in the ZH11 background. The expression of *Ghd7* was observed not affected at 2.5 h before dawn (Fig. 6a-c), while those downstream heading-date related genes, *Ehd1*, *Hd3a* and *RFT1* were differentially expressed at this time point in ZH11, *osprr37*, *osgi*, *phyB*, *hd1* and higher order double mutants (Fig. 6a-i). We believe that those effects could be explained by the previous finding that the repression of *Ehd1* by *Ghd7* depends on *Ghd7* expression levels on the previous morning (Itoh et al. 2010). We previously investigated the heading dates of several near-isogenic lines with a combination of either functional or defective alleles of *OsPRR37*, *Ghd7*, *Ghd8* and *Hd1* in the Zhenshan 97 background (Zhang et al. 2019a). *OsPRR37* was found consistently delays heading date regardless of other heading date genes under NLD conditions but exhibits a promoting effect under NSD conditions only in backgrounds with functional *Ghd7*. Therefore, the alternative promotion effect on heading date by *OsPRR37* is dependent on *Ghd7*.

### Upstream Signals from *OsGI* and *PhyB* are Essential for the Promotion Effect of *OsPRR37* on Heading Date

Knocking out either *OsGI* or *PhyB* in the *osprr37* background (*osprr37 osgi* and *osprr37 phyB*) promoted heading compared with *osprr37*, but the heading date of both double mutants was still later than that of the corresponding single mutants (Fig. 5i, j), which is consistent with the observation that the expression levels of *Ehd1*, *Hd3a* and *RFT1* in double mutants were intermediate between those of *osprr37* and the corresponding single mutants (Fig. 6d-l). However, the *phyB* line displayed an earlier heading date and a lower expression level of *Ghd7* than ZH11 (Fig. 6b), which is contrary to previous results showing that higher expression of *Ghd7* were detected in *phyB* compared with wild-type Nipponbare (Osugi et al. 2011). These different results could be attributed to the different tested tissues used because flag leaves were used here whereas the whole aboveground parts of plants were used in the previous study (Osugi et al. 2011). In addition, mutation of the circadian clock-related gene *OsGI* reduced the expression level of *Ghd7* (Fig. 6a), which is in agreement with a previous report (Itoh et al. 2010). Therefore, the promotion effect of *OsPRR37* on heading date requires an intact *Ghd7* regulatory pathway involving not only circadian clock signals transduced by *OsGI*, but also light signal perception via *PhyB*.

### Alternative Effect of *OsPRR37* is Genetic Background Dependent

Our results here and in our previous study (Zhang et al. 2019a) demonstrated the promotion effect on heading date by *OsPRR37* in different genetic backgrounds under different day-length conditions. Regardless of day-length conditions, *OsPRR37* consistently promotes heading date in the ZH11 background under either NLD or NSD conditions (Fig. 1b). However, *OsPRR37* exhibits completely different effects under NLD and NSD conditions in the Zhenshan 97 background (Fig. 3f-k). It promoted heading date under NSD conditions, but delayed heading date under NLD conditions. Although data of flowering-time under NSD and NLD in different years were not strictly comparable, it is also possible that an unknown daylength sensitive gene may exist and display functionality divergences in the ZH11 and Zhenshan97 backgrounds. Further investigation with a population deriving from crossing between ZH11 and near-isogenic line *OsPRR37 Ghd7 Ghd8 Hd1* in the Zhenshan 97 background may facilitate the isolation of the unknown gene. We also observed that promotion effect of *OsPRR37* partially depends on *Ghd7* in the ZH11 background (Fig. 4d) but completely relies on *Ghd7* in the Zhenshan 97 background (Zhang et al. 2019a). Thus, *OsPRR37* may act in another pathway independent of *Ghd7* to regulate heading date in ZH11.

## Conclusions

Through map-based cloning and the MutMap strategy, we cloned a gene heading date gene *LHD7* which is allelic to *OsPRR37*. Our results revealed the novel function of *OsPRR37* in the promotion of heading date in the ZH11 background under both NLD and NSD conditions, which is opposite to the previous finding that *OsPRR37* acts as a suppressor of heading date. Further genetic analysis demonstrated the promotion effect on heading date by *OsPRR37* was partially dependent on *Ghd7* and *Ghd7*-related pathway in the ZH11 background. Our finding not only revealed an alternative promotion function of *OsPRR37* in the regulation of heading date, but also enriches the theoretical bases for improvement of heading date of rice in the future.

## Supplementary Information

**Additional file 1: Table S1**. Primers used in this study.

**Additional file 2: Figure S1**. Expression levels of indicated genes in leaves of 40-d-old plants under controlled LD conditions were determined by quantitative real-time PCR (qRT-PCR) and shown as mean ± SD of three replicates. Rice ubiquitin gene (Os02g0161900) was used for normalization.

## Data Availability

The datasets supporting the conclusions of this article are included within the article and its additional files.

## Abbreviations

PR: Pseudo-receiver
PRR: Pseudo-response regulator
CCT: CONSTANS, CO-like, and TOC1
LD: Long day
SD: Short day
NLD: Natural long day
NSD: Natural short day
SNP: Single nucleotide polymorphism
KASP: Kompetitive allele specific PCR
CRIPSPR: Clustered regularly interspaced short palindromic repeats
