# Supplementary material for: OsPRR37 Alternatively Promotes Heading Date Through Suppressing the Expression of Ghd7 in the Japonica Variety Zhonghua 11 under Natural Long-Day Conditions
Source: Rice (N Y). 2021 Feb 25;14:20. doi: 10.1186/s12284-021-00464-1 (PMC7907330; doi:10.1186/s12284-021-00464-1)
Supplement: Supplementary file 1 — Additional file 1: Table S1. Primers used in this study. [file 12284_2021_464_MOESM1_ESM.docx]

**Table S1 Primers used in this study**

| Primer names | | Sequence of primers (5'-3') |  |
| --- | --- | --- | --- |
| Ghd7.1-UF | TTACGAACGATAGCCGGTACCATGATGGGAACCGCTCATCA | | |
| Ghd7.1-UR | TCTAGAGGATCCCCGGGTACCTCATCTGTCCGCTGCCGC | | |
| Ghd7-CRF | ATCCCCTGGCACGCACTCGGGTTTTAGAGCTAGAAATAGCAAGTTA | | |
| Ghd7-CRR | CCGAGTGCGTGCCAGGGGATgccacggatcatctgcacaac | | |
| OsGI-CRF | ACTGTTCTGGCCCCCACCACGTTTTAGAGCTAGAAATAGCAAGTTA | | |
| OsGI-CRR | GTGGTGGGGGCCAGAACAGTgccacggatcatctgcacaac | | |
| PhyB-CRF | GGCGGGCACATACAGCCCTTGTTTTAGAGCTAGAAATAGCAAGTTA | | |
| PhyB-CRR | AAGGGCTGTATGTGCCCGCCAACCTGAGCCTCAGCGCAGC | | |
| Hd1-CRF | AACGTGTTCGACCAGGAGGTGTTTTAGAGCTAGAAATAGCAAGTTA | | |
| Hd1-CRR | ACCTCCTGGTCGAACACGTTgccacggatcatctgcacaac | | |
| SNP-UBA-F | GAAGGTGACCAAGTTCATGCTTCAAGTCACTAAGAGATGCTATGAG | | |
| SNP-UBA-R | GAAGGTCGGAGTCAACGGATTTCAAGTCACTAAGAGATGCTATGAA | | |
| SNP-UBA-C | CAGAGCTTGAAATGCTAGGTGAAG | | |
| SNP-290-F | GAAGGTGACCAAGTTCATGCTgataggcacggctagttccc | | |
| SNP-290-R | GAAGGTCGGAGTCAACGGATTgataggcacggctagttcct | | |
| SNP-290-C | gatgagatcagagacaacgcaatg | | |
| SNP-WD-F | GAAGGTGACCAAGTTCATGCTGCATATAATACATGTGTATCTTGTAGGTATGG | | |
| SNP-WD-R | GAAGGTCGGAGTCAACGGATTGCATATAATACATGTGTATCTTGTAGGTATGA | | |
| SNP-WD-C | TCAGAAATGTCCCCTCTACAACTG | | |
| SNP-37-F | GAAGGTGACCAAGTTCATGCTACAGTTTTTAAGTGTTTGTCAAAGGG | | |
| SNP-37-R | GAAGGTCGGAGTCAACGGATTACAGTTTTTAAGTGTTTGTCAAAGGA | | |
| SNP-37-C | CACATGCTGCCATAGGTTCTTAAG | | |
| qEhd1-F | TGGAAATCTCGAAAAACCCG | | |
| qEhd1-R | GCGCTAGCAAAGCTTCGGT | | |
| qHd3a-F | gctcactatcatcatccagcatg | | |
| qHd3a-R | ccttgctcagctatttaattgcataa | | |
| qRFT1-F | tgacctagattcaaagtctaatcctt | | |
| qRFT1-R | tgccggccatgtcaaattaataac | | |
| qGhd7-F | aggtgctacgagaagcaaatcc | | |
| qGhd7-R | gggcctcatctcggcatag | | |
| qGhd7.1-F | AATGGAAGCAATGGGCAGAATGGG | | |
| qGhd7.1-R | TGATCACTGCAGCCACTCTATGCT | | |
| qGhd8-F | cgtgcaatggtttagactaaag | | |
| qGhd8-R | aacagcatcagcatcaacaa | | |
| qHd1-F | tcagcaacagcatatctttctcatca | | |
| qHd1-R | tctggaatttggcatatctatcacc | | |
| qOsGI-F | ATCGTTCTGCAGGCCGAGA | | |
| qOsGI-R | TCACCAATGCTTCTGGGCTAT | | |
| qOsELF3-F | TACTTCCCGCCTTTCAGCATACCA | | |
| qOsELF3-R | ATGAGATCCACGACTGCTGCTCAA | | |
| qOsMADS50-F | gaccgtaacatcaacaccac | | |
| qOsMADS50-R | gagatccagcttattcctgg | | |
| qOsMADS51-F | gaaatcaaagaagatgttggcaaa | | |
| qOsMADS51-R | cttcctcctgccccctagag | | |
| qOsMADS56-F | AGCTCTTGATGAGTCCAGACGGAA | | |
| qOsMADS56-R | AGTCCGCTCCTTCTCTTTCAGCTT | | |
| qOsCO3-F | GGAGAAGAGGAAGACGAGGC | | |
| qOsCO3-R | TAGCTAAGCAACCAAGATGTA | | |
| qCOL4-F | ATCCACTCGGCGAACCCGCT | | |
| qCOL4-R | CGCTTCTCCCTGTACCGCAT | | |
| qOsCOL10-F | CGCCCTCGCTTCGATCCCA | | |
| qOsCOL10-R | TCCCTCTCGCCGCCGGTCA | | |
| qOsCOL13-F | GAGTTCACTTCTGGGCATGGTG | | |
| qOsCOL13-R | TCAGTGCAATCGGTTGACATGA | | |
| qDTH2-F | CCAGTTTCAACGACGCCTAA | | |
| qDTH2-R | GTCTCCATATACGCTCCCATCA | | |
| qEhd2-F | AGCTGAAGAGAGTGCAAGGCTTCT | | |
| qEhd2-R | TGATGTTGCTGTTGTTGCAGGTGG | | |
| qEhd3-F | CAAACACTGTGGCACATGCGAAGA | | |
| qEhd3-R | TCATCCTTGTCTTGGAAGCACCCT | | |
| qEhd4-F | CAGCCAGCGGAATCATCAC | | |
| qEhd4-R | CCAAATCCATCAGACCTACTCCT | | |
| OsU6-F | CCCCTTTCGCCAGGGGTACCtatgtacagcattacgtagg | | |
| OsU6-R | TACGAATTCGAGCTCGGTACCgatggtgcttactgtttag | | |
| OsU3-F | CCCCTTTCGCCAGGGGTACCgtaattcatccaggtctccaag | | |
| OsU3-R | TACGAATTCGAGCTCGGTACCgctgtgccgtacgacggtacg | | |
