# Supplementary material for: OsPRR37 Alternatively Promotes Heading Date Through Suppressing the Expression of Ghd7 in the Japonica Variety Zhonghua 11 under Natural Long-Day Conditions
Source: Rice (N Y). 2021 Feb 25;14:20. doi: 10.1186/s12284-021-00464-1 (PMC7907330; doi:10.1186/s12284-021-00464-1)
Supplement: Supplementary file 2 — Additional file 2: Figure S1. Expression levels of indicated genes in leaves of 40-d-old plants under controlled LD conditions were determined by quantitative real-time PCR (qRT-PCR) and shown as mean ± SD of three replicates. Rice ubiquitin gene (Os02g0161900) was used for normalization. [file 12284_2021_464_MOESM2_ESM.docx]

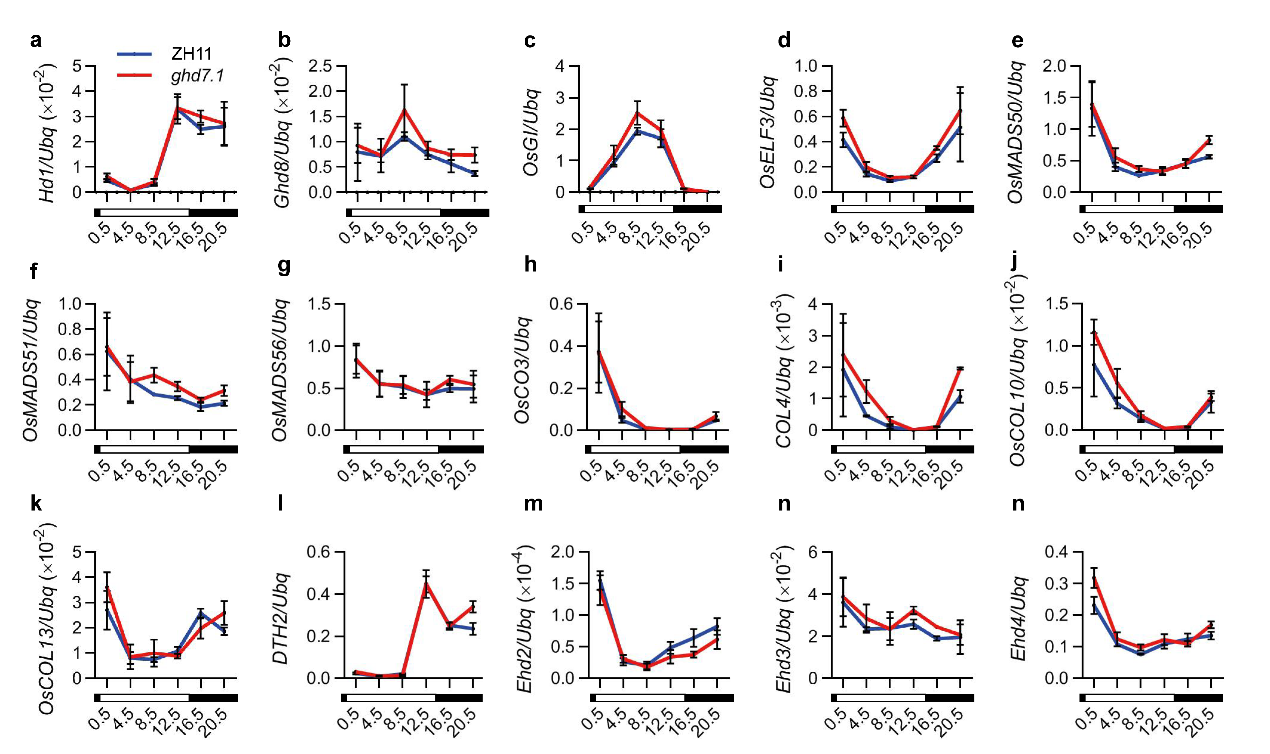


**Figure S1** Expression levels of indicated genes in leaves of 40-d-old plants under controlled LD conditions were determined by quantitative real-time PCR (qRT-PCR) and shown as mean ± SD of three replicates. Rice ubiquitin gene (Os02g0161900) was used for normalization.
